# Supplementary figures and images for: RNA sequencing of CD4 T-cells reveals the relationships between lncRNA-mRNA co-expression in elite controller vs. HIV-positive infected patients
Source: PeerJ. 2020 Apr 21;8:e8911. doi: 10.7717/peerj.8911 (PMC7182024; doi:10.7717/peerj.8911)

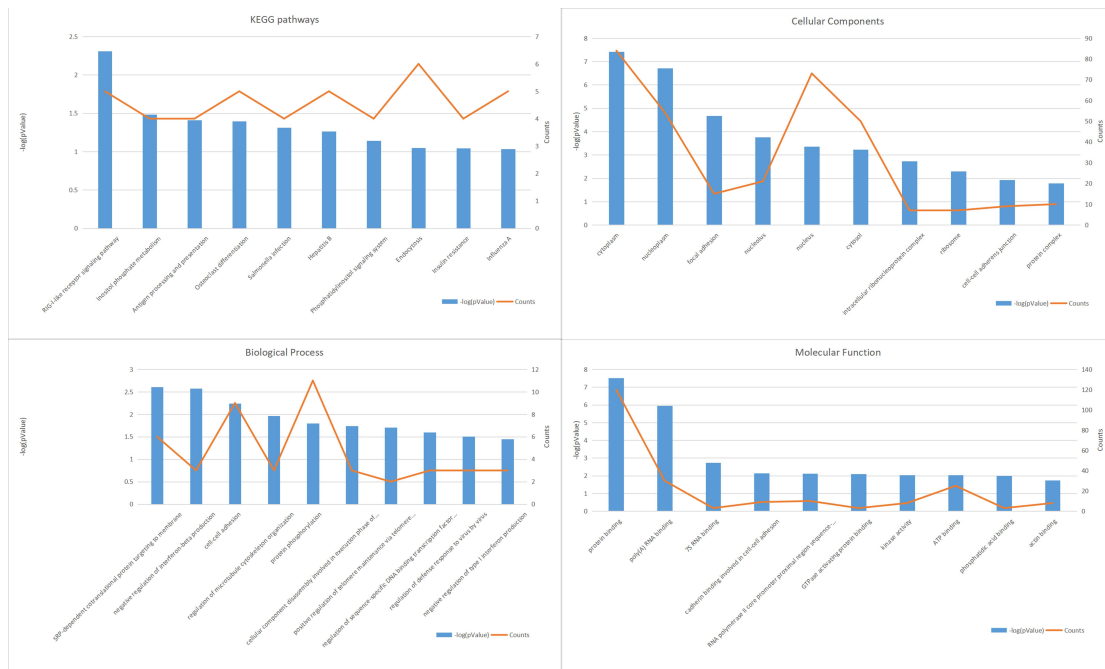

Supplement: Figure S1 — The height of the histogram correlates positively with the significance of the annotation, and the line graph represents the number of genes enriched under the annotation. [file peerj-08-8911-s001.pdf]

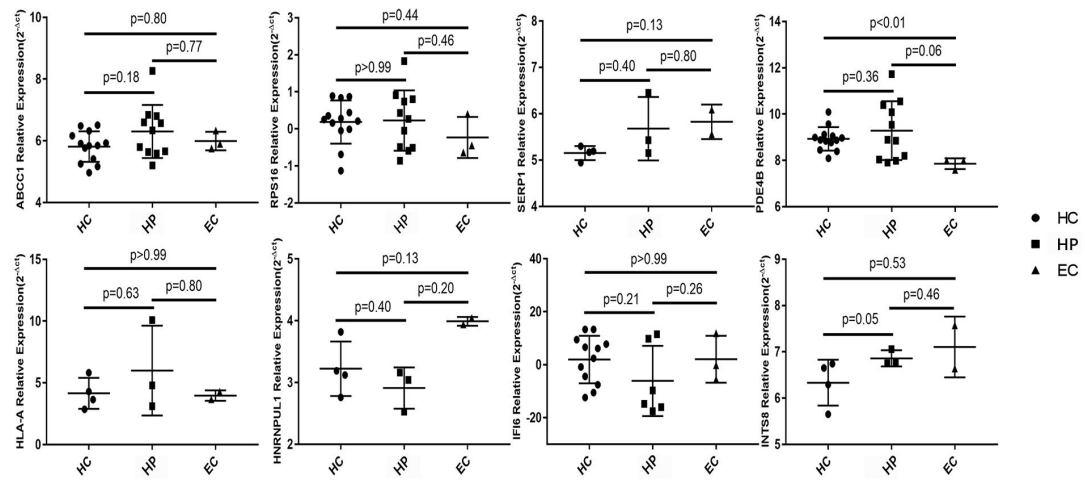

Supplement: Figure S2 [file peerj-08-8911-s002.pdf]

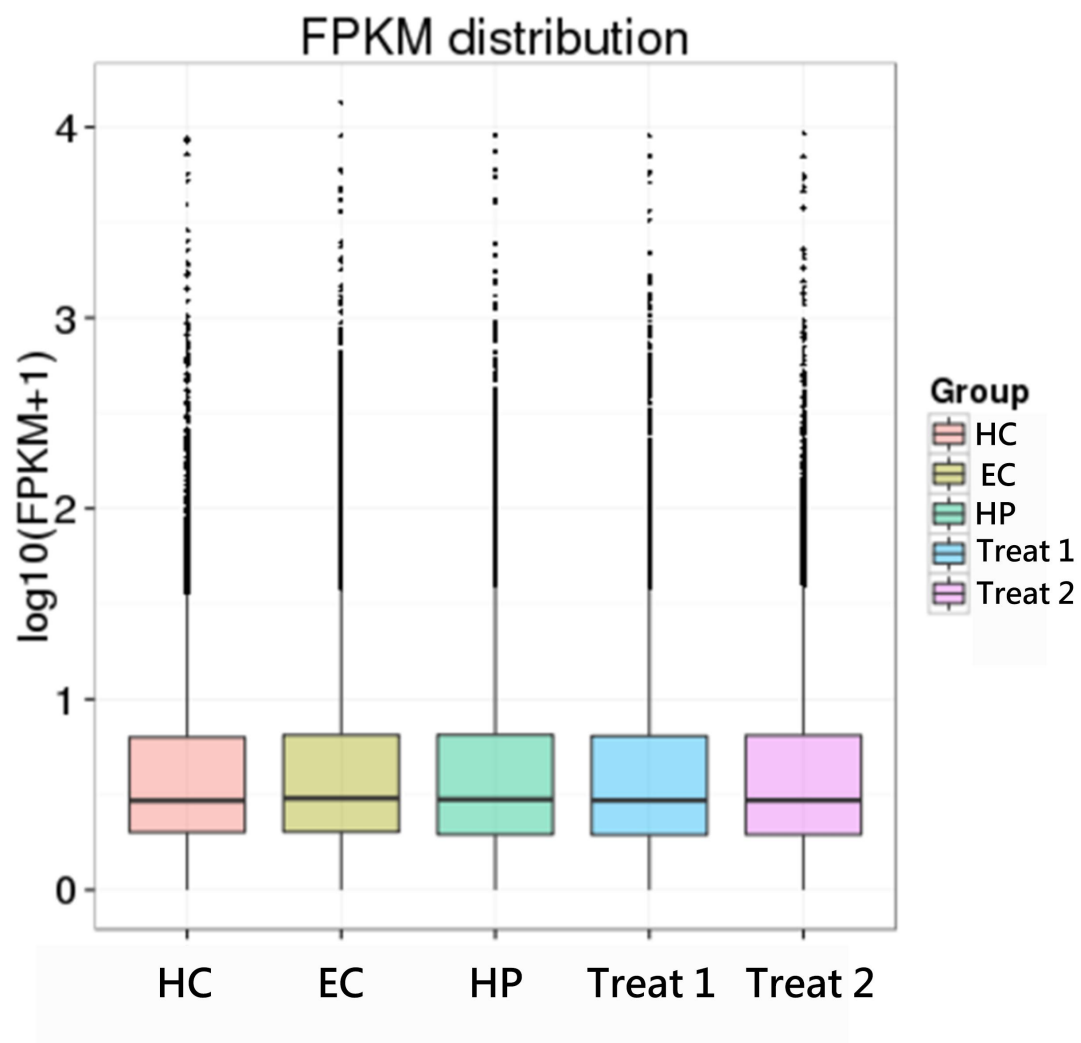

Supplement: Figure S3 — Horizontal coordinates show the group names, the ordinate shows the log10 (FPKM-1), each area of the box map corresponds to five statistics (top to bottom are the maximum, top quartile, median, down quartile, and minimum value). [file peerj-08-8911-s003.pdf]

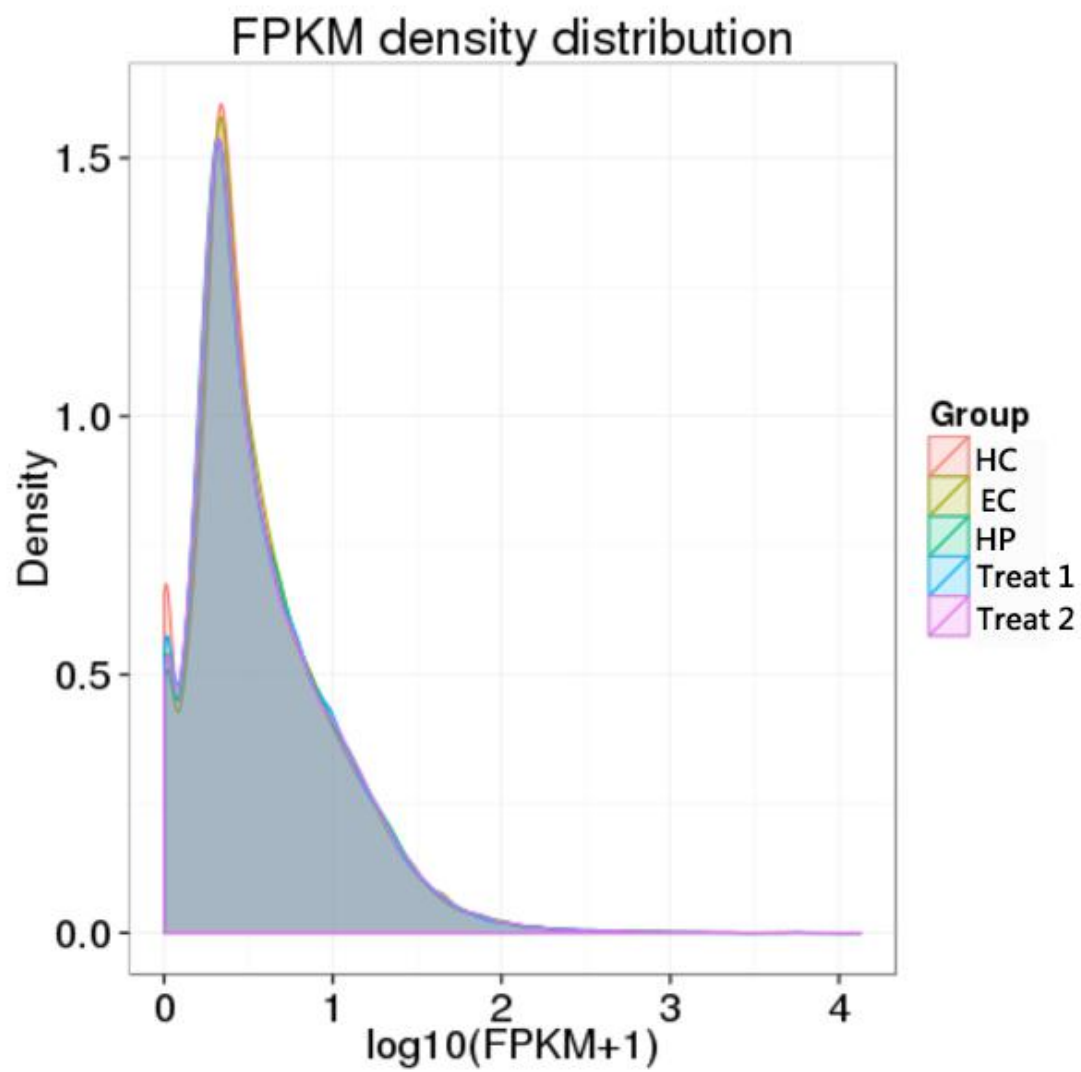

Supplement: Figure S4 — Horizontal coordinates of log10 (FPKM-1) and the ordinate showing the density of the genes. Table S1. Clinical characteristics of the participants. [file peerj-08-8911-s004.pdf]
